# A few final thoughts as outgoing editor-in-chief

**DOI:** 10.1186/s42155-025-00598-z

**Published:** 2025-09-01

**Authors:** Jim A. Reekers

**Affiliations:** AmsterdamUMC, University of Amsterdam, Amsterdam, The Netherlands

Launching successfully a new medical journal is not easy and requires a lot of time and hard work. That is one of the things I have learned during my eight years as editor-in-chief. One of the problems is finding your own voice within the IR community in order to carve out a niche alongside the long-established CVIR journal. It was therefore essential from the very start to identify a new target audience. IR is based on constant innovation and the discovery of new treatment options. This important practical side of IR is often seen in case reports and small studies that rapidly provide information for daily practice. That is why I have focused on building a journal with practical information that can be used immediately in daily practice. Scientific research is less prominent in the journal. Although this focus has a negative effect on the impact factor due to a lower number of citations, the journal currently has a respectful impact factor of 1.5. I am extremely proud of this.

The online open access format is an essential tool for the rapid disseminating of new information. I predict that within a few years, all medical journals will be online and open access. However, this format has a major disadvantage, namely the publication fee that authors have to pay. Certainly, for journals with a lower impact factor and a smaller audience, it is not attractive for authors to pay for every case report or small study. A lot of potentially valuable information will be lost as a result. It wouldn’t be a bad idea for publishers to follow the model used in the music industry. A streaming service allows you to listen to all music via a monthly subscription. You pay an extra fee to download songs. The same principle could be applied to all medical publications that are then accessible through read only via a subscription. The monthly subscription price could vary and be based on the number of views per month. Additionally, it would still be possible to publish open access via a publication fee. I am convinced that this could also be an attractive revenue model for publishers.

A medical journal is a living archive that doctors use to keep up to date with the latest developments in their field. The new knowledge is used immediately to treat patients. This has implications for the impartiality and honesty of publications in medical journals. Fraudulent or biased publications lead to poor patient care. It is an unreasonable task for an editor-in-chief to review all submitted manuscripts for fraudulent and biased content. The editor-in-chief has an editorial board to help him, but his most important source of feedback are the journal’s reviewers. To provide readers of a journal with the best possible guarantee that every article has been thoroughly reviewed by external reviewers, it is essential that this process is fully transparent. Only then can readers trust the final publication in the journal. In most medical journals, this external review is traditionally conducted in secret, without any transparency. I am extremely pleased that CVIRendovascular has used a system of open peer review from the very beginning. The BMJ and an ever-increasing number of journals now use open peer review. I consider this to be one of the most important cornerstones of CVIRendovascular’s credibility and quality. This should never be lost!

A journal is only as good as its reviewers; they are the most important resource of any journal. We expect a lot from reviewers but the fact that they are not compensated in any way for their important work is a time-bomb under the current system. Reviewers should be compensated financially for their work. The extra bonus of this is that, as an editor, you can also introduce standards and certification for reviewers.

Finally, a personal note. I have greatly enjoyed working for CVIRendovascular over the past eight years. I thank the editorial board, the editors, and all the reviewers for their huge efforts. And last but not least I want to thank the editorial office in Vienna which did a tremendous job. Not only to support and stimulate me but also kept me on track when my ambition and plans started to lose contact with reality. Author video`s, visual abstracts and newsletters are a few examples of the outcome of this great cooperation. Together, we have made CVIRendovascular what it is today. It is with melancholy but also with pride that I hand over the journal to Rob Morgan as the new editor-in- chief, and I wish him every success in further developing this project. Goodbye.

